# Compulsory Citizenship Behavior, Work Stress, Perceived Quiet Firing, and Quiet Quitting Among Nurses: A Mediation and Exploratory Serial Mediation Analysis

**DOI:** 10.1155/jonm/1888100

**Published:** 2026-08-17

**Authors:** Hanife Tiryaki Şen, Demet Yurtsever, Şehrinaz Polat

**Affiliations:** ^1^ Hamidiye Faculty of Nursing, Department of Nursing Administration, University of Health Sciences, Istanbul, Türkiye, akdeniz.edu.tr; ^2^ Director of Healthcare Services, Kanuni Sultan Süleyman Training and Research Hospital, Istanbul, Türkiye, kanunieah.gov.tr; ^3^ Faculty of Nursing, Department of Nursing Administration, Istanbul University, Fatih, İstanbul, Türkiye, istanbul.edu.tr

**Keywords:** compulsory citizenship, nurse, quiet firing, quiet quitting, work stress

## Abstract

**Aim:**

This study aimed to identify factors associated with quiet quitting and perceived quiet firing among nurses and to examine the mediating role of work stress and the exploratory serial indirect association involving perceived quiet firing in the relationship between compulsory citizenship behavior and quiet quitting.

**Method:**

A cross‐sectional, model‐testing design was employed with 624 nurses from three public hospitals. Sample size was determined pragmatically by inviting all eligible nurses not on long‐term leave. Because all eligible nurses were invited during the study period, an a priori power analysis was not considered necessary. Instead, the sample size was pragmatically determined based on the accessible population. Statistical analyses included Pearson’s correlation, multivariate linear regression using the enter method, and regression‐based bootstrap mediation analyses, including simple mediation models and an exploratory PROCESS Model 6 serial mediation model with 5000 resamples and 95% bias‐corrected confidence intervals (BCIs).

**Results:**

Increases in general work stress and compulsory citizenship behavior were significantly associated with higher perceptions of quiet quitting and quiet firing. In addition, general work stress partially mediated the relationship between compulsory citizenship behavior and nurses’ perceptions of quiet quitting and quiet firing.

**Conclusion:**

These findings highlight the importance of improving nurses’ working conditions, ensuring alignment between duties and job descriptions, and encouraging active participation in decision‐making.

**Implication for Nursing Management:**

Nursing managers should address organizational conditions associated with work‐related stress and perceived involuntary resignation. Regular feedback mechanisms, workload monitoring, and inclusive decision‐making processes may help strengthen commitment and reduce disengagement among nurses.

## 1. Introduction

In organizational contexts, the metaphor of quiet is frequently used to describe phenomena reflecting the detrimental effects of workplace silence [1]. Quiet quitting (QQ) refers to employees limiting their efforts strictly to tasks within their formal job roles—remaining in position while deliberately reducing work‐related effort—and has reportedly become more prevalent since the COVID‐19 pandemic [2, 3]. Employees exhibiting QQ tendencies perform assigned duties at a minimal level [4, 5] and avoid responsibilities beyond their formal role descriptions [6].

Quiet firing (QF) is conceptually distinct from QQ and should be clearly differentiated: while QQ centers on employees’ voluntary behavioral choices, QF reflects organizationally or manager‐driven actions. QF refers to the indirect exclusion or marginalization of an employee without formal termination [7]. It includes behaviors ranging from withholding guidance, support, and career development opportunities to creating adverse work conditions that undermine employee confidence [7, 8]. In nursing, QF may manifest as denial of professional development, exclusion from decision‐making, or reassignment from preferred shifts [7].

Organizational demands exceeding employees’ defined roles can produce psychological strain and elevated work stress [9–11]. The job demands–resources (JD‐R) model posits that when job demands chronically exceed available resources, employees experience strain manifesting as stress, disengagement, or withdrawal [9]. Conservation of resources (COR) theory further suggests that employees experiencing sustained resource depletion—as arises from compulsory extra‐role demands—will attempt to protect remaining resources through behavioral strategies, including reduced discretionary effort [12].

Compulsory citizenship behavior (CCB) refers to involuntary, extra‐role behaviors performed in response to organizational pressure rather than personal initiative [12, 13]. Managers may expect nurses to engage in CCBs such as working late, assisting colleagues, or fulfilling work obligations during personal time [14]. Such pressured behaviors can induce stress and contribute to adverse employee outcomes [15]. Evidence also suggests CCBs are more prevalent in Eastern cultural contexts [12, 16].

Although QQ and QF have received increasing scholarly attention, they remain less extensively studied than other work‐related constructs [2]. Research examining these phenomena among healthcare professionals remains limited [17]. The present study examined the associations among CCB, QQ, QF, and work stress and evaluated simple mediation and exploratory serial mediation models using cross‐sectional data.

 H1: CCB is positively associated with QQ, perceived QF, and work stress.
 H2: work stress is positively associated with QQ and perceived QF and statistically mediates the associations between CCB and both outcomes.
 H3 (exploratory): CCB is associated with QQ through an exploratory serial indirect pathway involving perceived QF and work stress (CCB ⟶ QF ⟶ work stress ⟶ QQ).

## 2. Methods

### 2.1. Aim

The aim of this study was to examine the relationships between nurses’ CCBs and their perceptions of QQ and QF and to evaluate the mediating role of work stress in these relationships.

### 2.2. Design

This study adopted a model‐testing approach using cross‐sectional data. The cross‐sectional design precludes causal inference; all findings should be interpreted as associations rather than causal effects.

### 2.3. Population and Sample

The study population consisted of 1390 nurses from three public hospitals. All eligible nurses not on long‐term leave were invited to participate (*n* = 827). Of these, 636 agreed; 12 were excluded for incomplete responses. The final sample comprised 624 nurses (acceptance rate: 76.9%; response rate: 98.1%). Sample size was determined pragmatically by inviting all eligible nurses. Post hoc justification: simulation studies indicate that *N* ≥ 200 provides adequate power (≥ 0.80) to detect indirect effects of the observed magnitude [18]; *N* = 624 substantially exceeds this threshold.

Inclusion criteria: active employment and willingness to provide informed consent. Exclusion criterion: long‐term leave. Both nurse managers and staff nurses were included, as both groups are subject to CCB pressures in clinical settings.

### 2.4. Data Collection Process

Data were collected between November 30 and December 30, 2024, via face‐to‐face questionnaire administration. Written informed consent was obtained from all participants.

### 2.5. Data Collection Instruments

QQ Scale: developed by Anand et al. [19] and adapted into Turkish by Tiryaki Sen et al. [20]. Seven items, five‐point Likert scale (1–5); scores 7–35. Higher scores = greater tendency toward QQ. Cronbach’s *α*: original 0.82, Turkish 0.89, present study 0.71.

QF Scale: developed by Anand et al. [19] and adapted into Turkish by Tiryaki Sen et al. [20]. Seven items, five‐point Likert scale (1–5); scores 7–35. Higher scores = stronger perceptions of QF. Cronbach’s *α*: original 0.87, Turkish 0.92, present study 0.87.

CCB Scale: developed by Vigoda‐Gadot [13] and adapted into Turkish by Harmancı Seren and Ünaldı Baydın [21]. Five items, five‐point Likert scale; scores 5–25. Cronbach’s *α*: original 0.83, Turkish 0.88, present study 0.91.

General Work Stress Scale (GWSS): developed by De Bruin [22] and adapted into Turkish by Teleş [23]. Nine items, five‐point Likert scale; scores 9–45. Higher scores = higher work stress. Cronbach’s *α*: Turkish 0.91, present study 0.93.

### 2.6. Data Analysis

Statistical analyses were performed using SPSS Version 26. Normality was evaluated via skewness and kurtosis coefficients, with values within ±1.5 considered acceptable. Internal consistency was assessed using Cronbach’s *α*. Descriptive statistics included frequencies, percentages, means, and standard deviations. Pearson’s correlation analysis examined bivariate associations among the study variables. Multivariate linear regression analyses using the enter method, with all predictors entered simultaneously, were conducted to identify variables independently associated with QQ (Model A) and perceived QF (Model B). Reference categories for categorical variables are specified in table footnotes. Multicollinearity was assessed using tolerance and variance inflation factor (VIF) values. Although age and professional experience were highly correlated, diagnostic values remained within acceptable limits; therefore, both variables were retained to allow comprehensive covariate adjustment. Simple mediation analyses were conducted using regression‐based bootstrap procedures with 5000 resamples and 95% bias‐corrected confidence intervals (BCIs). Work stress was examined as a mediator in the associations between CCB and QQ and between CCB and perceived QF. In addition, an exploratory serial mediation analysis was conducted using PROCESS Model 6 to examine whether CCB was associated with QQ through perceived QF and work stress. As a sensitivity check, the serial model was repeated controlling for age, gender, and work schedule, yielding substantively unchanged results. Given the cross‐sectional design, all mediation findings were interpreted as statistical indirect associations rather than evidence of causal mechanisms. Statistical significance was set at *p* < 0.05 (two‐sided).

### 2.7. Ethical Considerations

Ethical approval for the study was obtained from the Istanbul Kanuni Sultan Süleyman Training and Research Hospital Scientific Research Ethics Committee (Date: 30.10.2024, Approval Number: 228). Following this approval, written permission was obtained from the administrations of the three hospitals where the study was conducted. Permission to use the measurement scales was obtained from the original authors via email. All nurses participated voluntarily and provided written informed consent prior to data collection. The study was conducted in accordance with the ethical principles outlined in the Declaration of Helsinki.

## 3. Results

### 3.1. Descriptive Characteristics and Bivariate Associations

The study included 624 nurses with a mean age of 30.14 ± 6.58 years and a mean professional experience of 7.22 ± 6.83 years. Most participants were female (80.1%), single (54.0%), and held a bachelor’s degree (76.9%). The majority worked as staff nurses (89.1%) and on rotating shifts (70.4%). Regarding clinical units, 37.0% worked in inpatient wards and 29.2% in intensive care units, whereas smaller proportions were employed in emergency departments (12.3%), outpatient units (11.1%), operating rooms (5.6%), and other clinical settings (4.8%).

The mean QQ score was 16.79 ± 5.26 (range: 7–35), the perceived QF score was 17.43 ± 6.57 (range: 7–35), the general work stress score was 26.26 ± 9.55 (range: 9–45), and the CCB score was 16.31 ± 5.55 (range: 5–25). Pearson correlation analyses demonstrated significant positive associations between QQ and perceived QF (*r* = 0.584, *p* < 0.001), general work stress (*r* = 0.571, *p* < 0.001), and CCB (*r* = 0.492, *p* < 0.001). Perceived QF was also positively associated with general work stress (*r* = 0.557, *p* < 0.001) and CCB (*r* = 0.547, *p* < 0.001). Furthermore, general work stress was strongly correlated with CCB (*r* = 0.689, *p* < 0.001).

### 3.2. Multivariate Regression Analysis (Enter Method)

Multivariate linear regression analyses showed that both regression models were statistically significant. The model predicting QQ explained 37.2% of the variance, while the model predicting perceived QF explained 38.7% of the variance. No severe multicollinearity was detected (all VIFs < 7; tolerances > 0.10); however, age and professional experience showed elevated VIF values due to their high intercorrelation. Durbin–Watson statistics indicated no autocorrelation.

In Model A, male gender, rotating shifts, general work stress, and CCB were independently associated with higher QQ. Age, marital status, education, position, and professional experience were not independently associated with QQ. Detailed regression coefficients, reference categories, tolerance values, VIF values, and model summaries are presented in Table 1.

**TABLE 1:** Variables associated with quiet quitting, multivariate linear regression, and enter method (Model A).

Model A	*B*	SE	*β*	*t*	*p*	Tolerance	VIF
Constant	3.647	1.283	—	2.842	0.005		

Age	0.069	0.064	0.086	1.080	0.281	0.161	6.221
Male gender (ref: female)	1.075	0.433	0.082	2.484	0.013	0.946	1.057
Marital status	0.107	0.396	0.010	0.270	0.787	0.724	1.380
Education	−0.273	0.273	−0.033	−1.002	0.317	0.922	1.085
Position	−0.284	0.586	−0.017	−0.484	0.628	0.844	1.184
Professional experience (years)	−0.065	0.058	−0.084	−1.111	0.267	0.178	5.628
Rotating shifts (ref: day shift)	1.467	0.491	0.128	2.990	0.003	0.561	1.781
General work stress	0.233	0.025	0.424	9.430	< 0.001	0.505	1.981
Compulsory citizenship behavior	0.154	0.042	0.163	3.648	< 0.001	0.512	1.954

Model summary	Method = Enter | *F* (9, 614) = 40.455; *p* < 0.001 | *R* ^2^ = 0.372 | Durbin‐Watson = 1.846 | Dependent variable: Quiet Quitting						

*Note:* B = unstandardized coefficient; *β* = standardized coefficient. Reference categories: Gender = female; work schedule = day shift. Marital status, education, and position were entered according to the study coding used in the dataset. Age and professional experience showed elevated VIF values due to high intercorrelation (*r* = 0.903); no severe multicollinearity was detected (all VIFs < 7).

Abbreviations: SE = standard error; VIF = variance inflation factor.

In Model B, male gender, rotating shifts, general work stress, and CCB were independently associated with higher perceived QF. Age, marital status, education, position, and professional experience were not independently associated with perceived QF. Detailed regression coefficients, reference categories, tolerance values, VIF values, and model summaries are presented in Table 2.

**TABLE 2:** Variables associated with perceived quiet firing, multivariate linear regression, and enter method (Model B).

Model B	*B*	SE	*β*	*t*	*p*	Tolerance	VIF
Constant	−1.042	1.584	—	−0.658	0.511		

Age	0.123	0.079	0.123	1.560	0.119	0.161	6.221
Male gender (ref: female)	1.339	0.534	0.081	2.506	0.013	0.946	1.057
Marital status	−0.779	0.489	−0.059	−1.592	0.112	0.724	1.380
Education	−0.373	0.337	−0.036	−1.107	0.269	0.922	1.085
Position	0.116	0.725	0.005	0.160	0.873	0.844	1.184
Professional experience (years)	−0.004	0.072	−0.004	−0.056	0.955	0.178	5.628
Rotating shifts (ref: day shift)	2.040	0.606	0.142	3.366	< 0.001	0.561	1.781
General work stress	0.240	0.031	0.349	7.852	< 0.001	0.505	1.981
Compulsory citizenship behavior	0.349	0.052	0.294	6.662	< 0.001	0.512	1.954

Model summary	Method = enter | *F*(9, 614) = 43.112; *p* < 0.001 | *R* ^2^ = 0.387 | Durbin–Watson = 1.765 | Dependent variable: perceived quiet firing						

*Note:* B = unstandardized coefficient; *β* = standardized coefficient. Reference categories: Gender = female; work schedule = day shift. Marital status, education, and position were entered according to the study coding used in the dataset. Age and professional experience showed elevated VIF values due to high intercorrelation (*r* = 0.903); no severe multicollinearity was detected (all VIFs < 7).

Abbreviations: SE = standard error; VIF = variance inflation factor.

### 3.3. Simple Mediation Analysis (Models A and B)

Bootstrap mediation analyses indicated that work stress showed a statistically significant indirect association in both models. Specifically, CCB was associated with QQ and perceived QF partly through higher work stress. In both models, the direct associations remained statistically significant after work stress was included, indicating a pattern consistent with partial statistical mediation. Detailed path estimates, bootstrap confidence intervals, and direct and indirect effects are presented in Table 3.

**TABLE 3:** Bootstrap mediation results, work stress as a mediator (simple mediation).

Regression path	*B*	SE	95% BCI [LL, UL]	*p*
*Panel A: CCB ⟶ work stress ⟶ quiet quitting*				
Total effect (c): CCB ⟶ QQ	0.466	0.036	[0.393, 0.537]	< 0.001
Path a: CCB ⟶ work stress	1.187	0.048	[1.091, 1.282]	< 0.001
Path b: work stress ⟶ QQ	0.243	0.026	[0.190, 0.294]	< 0.001
Direct effect (c′): CCB ⟶ QQ	0.178	0.043	[0.091, 0.262]	< 0.001
Indirect effect (a × b): CCB ⟶ WS ⟶ QQ	0.288	0.034	[0.223, 0.356]^∗^	< 0.001
Partial mediation pattern (c′ significant, c′ < c)				

*Panel B: CCB ⟶ work stress ⟶ perceived quiet firing*				
Total effect (c): CCB ⟶ QF	0.648	0.040	[0.563, 0.734]	< 0.001
Path a: CCB ⟶ work stress	1.187	0.048	[1.091, 1.282]	< 0.001
Path b: work stress ⟶ QF	0.236	0.031	[0.174, 0.297]	< 0.001
Direct effect (c′): CCB ⟶ QF	0.368	0.054	[0.261, 0.473]	< 0.001
Indirect effect (a × b): CCB ⟶ WS ⟶ QF	0.280	0.038	[0.205, 0.354]^∗^	< 0.001
Partial mediation pattern (c′ significant, c′ < c)				

*Note:* QF = perceived quiet firing; BCI = bias‐corrected confidence interval; *B* = unstandardized coefficient. Bootstrap resamples = 5000.

Abbreviations: CCB = compulsory citizenship behavior; QQ = quiet quitting; SE = standard error; WS = work stress.

^∗^95% BCI excludes zero.

### 3.4. Exploratory Serial Mediation Analysis (PROCESS Model 6)

The exploratory serial mediation analysis showed that all three indirect pathways from CCB to QQ were statistically significant. These findings suggest that CCB may be associated with QQ through perceived QF, work stress, and the sequential indirect pathway involving perceived QF followed by work stress. The direct association between CCB and QQ was no longer statistically significant after both mediators were included. However, because the data were cross‐sectional, this pattern should be interpreted as an associative indirect pathway rather than as evidence of a causal or temporal mechanism. Sensitivity analyses controlling for age, gender, and work schedule yielded substantively unchanged results. Detailed PROCESS Model 6 estimates and bootstrap confidence intervals are provided in Table 4.

**TABLE 4:** Exploratory serial mediation bootstrap results, PROCESS Model 6 model: CCB ⟶ quiet firing (M1) ⟶ work stress (M2) ⟶ quiet quitting (Y).

Regression path	*B*	SE	95% BCI [LL, UL]	*p*
*Regression path coefficients*				
Path a1: CCB ⟶ quiet firing	0.648	0.040	[0.570, 0.726]	< 0.001
Path a2: CCB ⟶ work stress (Equation 2, controlling M1)	0.946	0.057	[0.834, 1.058]	< 0.001
Path d21: quiet firing ⟶ work stress (controlling X)	0.373	0.048	[0.278, 0.467]	< 0.001
Path b1: quiet firing ⟶ QQ (controlling X, M2)	0.295	0.030	[0.235, 0.354]	< 0.001
Path b2: work stress ⟶ QQ (controlling X, M1)	0.174	0.024	[0.127, 0.221]	< 0.001
Direct effect (c’): CCB ⟶ QQ (controlling M1, M2)	0.069	0.041	[−0.012, 0.149]	0.094
Total effect (c): CCB ⟶ QQ	0.466	0.033	[0.401, 0.531]	< 0.001

*Bootstrap indirect effects (5000 resamples)*				
Indirect 1: CCB ⟶ QF ⟶ QQ	0.191	0.026	[0.142, 0.242]	Significant^†^
Indirect 2: CCB ⟶ QF ⟶ WS ⟶ QQ (serial)	0.042	0.010	[0.025, 0.064]	Significant ^†^
Indirect 3: CCB ⟶ WS ⟶ QQ	0.164	0.021	[0.112, 0.220]	Significant^†^

Total indirect effect	0.397	0.035	[0.330, 0.469]	Significant ^†^

*Note:* Path coefficients are reported according to Hayes (2022) PROCESS Model 6 specification. Given the cross‐sectional design, serial mediation findings should be interpreted as associative rather than causal patterns.

^†^ *p* < 0.001.

## 4. Discussion

This study examined associations among QQ, QF, CCB, and work stress. Nurses reported moderate QQ (16.79 ± 5.26) and QF (17.43 ± 6.57), above‐moderate work stress (26.26 ± 9.55), and high CCB (16.31 ± 5.55), consistent with prior findings [7, 24, 25].

The association between CCB and higher work stress aligns with JD‐R theory: compulsory extra‐role demands intensify job demands without compensatory resource increases, producing strain. This is consistent with evidence from manufacturing [26], banking [27], and nursing contexts [28]. Higher work stress was in turn associated with greater QQ and QF perceptions, corroborating findings that stress, injustice, and burnout are linked to QQ [29, 30].

The simple mediation models indicated a partial statistical mediation pattern: work stress partly, but not fully, accounted for the CCB–QQ and CCB–QF associations. This suggests that CCB may also be linked to these outcomes through additional psychosocial or organizational pathways consistent with organizational pressure and alienation [31–33].

The exploratory serial mediation analysis provided additional insight into the pattern of associations among the study variables. The nonsignificant direct association between CCB and QQ after the inclusion of perceived QF and work stress, together with statistically significant indirect pathways, suggests that the relationship may operate through multiple interrelated psychosocial processes. In conceptual terms, compulsory extra‐role pressure may be associated with stronger perceptions of organizational marginalization and higher work stress, which in turn may be linked to greater QQ tendencies. However, this pattern should be interpreted as an exploratory associative pathway rather than a causal or temporal mechanism, given the cross‐sectional design. Longitudinal research is needed to determine whether these variables unfold in the proposed sequence over time. Although age and professional experience were not independently associated with QQ or QF in the enter‐method regression, their high intercorrelation likely reduced the individual predictive precision of each variable; both were retained to ensure comprehensive covariate adjustment. Sensitivity analyses indicated that the indirect associations remained substantively unchanged after controlling for age, gender, and work schedule.

Male gender and rotating shifts were each associated with higher QQ and QF, consistent with evidence that demanding work schedules contribute to adverse employee outcomes [2, 34].

### 4.1. Limitations

The cross‐sectional design precludes causal inference; findings should be interpreted as associations. The sequential patterns identified in the serial mediation model await validation in longitudinal designs. Self‐report data introduce common method bias risk. The limited number of comparable nursing studies restricts the depth of discussion. The sample was drawn from three public hospitals in one city, limiting generalizability.

### 4.2. Strengths

This study has several strengths. First, it addresses QQ and perceived QF among nurses, two emerging but still underexamined concepts in healthcare management research. Second, the study included a relatively large sample of nurses from three public hospitals, allowing for the examination of multiple organizational and individual variables. Third, the use of multivariate regression models enabled adjustment for key demographic and occupational covariates. Fourth, the regression‐based bootstrap mediation analyses provided a more detailed examination of the indirect associations linking CCB, work stress, perceived QF, and QQ. Finally, the study contributes to nursing management literature by distinguishing employee‐driven disengagement from perceived organization‐driven marginalization.

## 5. Conclusion

Nurses’ perceptions of QQ and perceived QF were at moderate levels, work stress was above moderate, and CCB was high. Work stress showed a statistically significant partial indirect association in the relationships between CCB and both QQ and perceived QF. The exploratory serial mediation analysis further suggested that CCB may be associated with QQ through perceived QF and work stress. However, because the study was cross‐sectional, these findings should be interpreted as associative rather than causal. Nursing managers should address compulsory extra‐role demands, workload pressure, and organizational practices that may contribute to stress, perceived marginalization, and disengagement. Future longitudinal and qualitative studies are needed to examine the temporal ordering and contextual meaning of these relationships.

## Author Contributions

The conception and design of the study: Hanife Tiryaki Şen, Demet Yurtsever, and Şehrinaz Polat.

Acquisition of data: Hanife Tiryaki Şen, Demet Yurtsever, and Şehrinaz Polat.

Analysis and interpretation of data: Demet Yurtsever, Hanife Tiryaki Şen, and Şehrinaz Polat.

Drafting the article: Hanife Tiryaki Şen, Demet Yurtsever, and Şehrinaz Polat.

Revising it critically for important intellectual content: Hanife Tiryaki Şen, Demet Yurtsever, and Şehrinaz Polat.

## Funding

This research did not receive any specific grant from funding agencies in the public, commercial, or not‐for‐profit sectors.

## Disclosure

This study has not been published elsewhere before.

## Ethics Statement

The study was approved by the Istanbul Kanuni Sultan Süleyman Training and Research Hospital Scientific Research Ethics Committee, dated 30.10.2024 and numbered 228.

## Conflicts of Interest

The authors declare no conflicts of interest.

## Supporting Information

Additional supporting information can be found online in the Supporting Information section.

## Supporting information


**Supporting Information** STROBE‐checklist‐v4‐combined‐PlosMedicine.

## Data Availability

The data that support the findings of this study are available from the corresponding author upon reasonable request.

## References

[1] Demirkaya H. , Yıldız B. , Özalcin S. E. , and Öztürk H. , Echoes of Silence in Human Resources: The Anatomy of Quiet Quitting in Organizations, Süleyman Demirel University Journal of Human Resource Management. (2023) 2, no. 2, 69–88.

[2] Galanis P. , Katsiroumpa A. , Vraka I. et al., The Quiet Quitting Scale: Development and Initial Validation, AIMS Public Health. (2023) 10, no. 4, 828–848, 10.3934/publichealth.2023055. 38187899 PMC10764970

[3] Hamouche S. , Koritos C. , and Papastathopoulos A. A. , Quiet Quitting: Relationship With Other Concepts and Implications for Tourism and Hospitality, International Journal of Contemporary Hospitality Management. (2023) 35, no. 12, 4297–4312, 10.1108/IJCHM-11-2022-1362.

[4] Harter J. , Is Quiet Quitting Real?, Gallup. (2022) https://www.gallup.com/workplace/398306/quiet-quitting-real.aspx.

[5] Formica S. and Sfodera F. , The Great Resignation and Quiet Quitting Paradigm Shifts: An Overview of Current Situation and Future Research Directions, Journal of Hospitality Marketing & Management. (2022) 31, no. 8, 899–907, 10.1080/19368623.2022.2136601.

[6] Thapa A. , How Quiet Quitting Became the Next Phase of the Great Resignation, CNBC. (2022) https://www.cnbc.com/2022/09/02/how-quiet-quitting-became-the-next-phase-of-the-great-resignation.html.

[7] Othman A. A. , Mahran H. M. , and Ali H. I. , Covering Loyalty Policy in Quiet Firing Workplace: The Association Between Quiet Quitting, Intention to Leave, and Nurses’ Loyalty, BMC Nursing. (2025) 24, no. 1, 1–11, 10.1186/s12912-025-03301-8. 40542367 PMC12181833

[8] Atta M. H. R. , Elzohairy N. W. , Abd Elaleem A. E. D. M. H. et al., Comprehending the Disruptive Influence of Workplace Gaslighting Behaviours and Mobbing on Nurses’ Career Entrenchment: A Multi-Centre Inquiry, Journal of Advanced Nursing. (2025) 81, no. 4, 1815–1828, 10.1111/jan.16368. 39113220

[9] Huang J. , Li Y. , and Tang C. , Leader’s Desire for Promotion, Workplace Anxiety and Exploitative Leadership: The Moderating Effect of Machiavellianism, Career Development International. (2023) 28, no. 6/7, 706–720, 10.1108/cdi-10-2022-0292.

[10] Zhang Q. , Dai W. , Chen J. , Gu Y. , and Zhao Y. , The ‘Side Effects’ of Digitalization: A Study on Role Overload and Job Burnout of Employees, PLoS One. (2025) 20, no. 4, 10.1371/journal.pone.0322112. PMC12043186 40305484

[11] Tang W. G. and Vandenberghe C. , Role Overload and Work Performance: The Role of Psychological Strain and Leader–Member Exchange, Frontiers in Psychology. (2021) 12, 10.3389/fpsyg.2021.691207. PMC8176030 34093378

[12] Yildiz B. , Kaptan Z. , Yildiz T. , Elibol E. , Yildiz H. , and Ozbilgin M. , A Systematic Review and Meta-Analytic Synthesis of the Relationship Between Compulsory Citizenship Behaviors and Its Theoretical Correlates, Frontiers in Psychology. (2023) 14, 10.3389/fpsyg.2023.1120209. PMC10192750 37213371

[13] Vigoda-Gadot E. , Redrawing the Boundaries of OCB? an Empirical Examination of Compulsory Extra-Role Behavior in the Workplace, Journal of Business and Psychology. (2007) 21, no. 3, 377–405, 10.1007/s10869-006-9034-5.

[14] Shaheen K. , Bashir M. , Shabbir R. , and Saleem S. , An Investigation of the Relationship Between Compulsory Citizenship Behaviour and Psychological Withdrawal, European Online Journal of Natural and Social Sciences. (2019) 8, no. 2, 331–343.

[15] Zhang Y. , Liu X. , Xu S. , Yang L.-Q. , and Bednall T. C. , Why Abusive Supervision Impacts Employee OCB and CWB: A Meta-Analytic Review of Competing Mediating Mechanisms, Journal of Management. (2019) 45, no. 6, 2474–2497, 10.1177/0149206318823935.

[16] Chen M. and Gao X. , Psychological Mechanism Between Impression Management and Compulsory Citizenship Behavior of Employees, Revista Argentina de Clinica Psicologica. (2020) 29, no. 1, 141–148, 10.24205/03276716.2020.19.

[17] Gün İ. , Evaluation of Factors Affecting Quiet Quitting Intention With AHP Method: An Application in Healthcare Workers, Journal of Administrative Sciences. (2024) 22, no. 52, 10.35408/comuybd.1405013.

[18] Hayes A. F. , Counterfactual/Potential Outcomes Causal Mediation Analysis With Treatment by Mediator Interaction Using PROCESS, Canadian Centre for Research Analysis and Methods Technical Report. (2022) 1–12.

[19] Anand A. , Doll J. , and Ray P. , Drowning in Silence: A Scale Development and Validation of Quiet Quitting and Quiet Firing, International Journal of Organizational Analysis. (2023) 32, no. 4, 721–743, 10.1108/IJOA-01-2023-3600.

[20] Tiryaki Sen H. , Yurtsever D. , and Polat Ş. , Validity and Reliability Study of “Quiet Quitting and Quiet Firing Scales” in Turkish: A Study on Nurses, Journal of Health and Nursing Management. (2024) 11, no. 2, 344–353, 10.54304/SHYD.2024.90187.

[21] Harmancı Seren A. K. and Ünaldı Baydın N. , Validity and Reliability Study of the Compulsory Citizenship Behaviour Scale in Turkish: A Study Among Nurses, Journal of Health and Nursing Management. (2017) 4, no. 2, 43–49, 10.5222/SHYD.2017.043.

[22] De Bruin G. P. , The Dimensionality of the General Work Stress Scale: A Hierarchical Exploratory Factor Analysis, SA Journal of Industrial Psychology. (2006) 32, no. 4, 68–75, 10.4102/sajip.v32i4.250.

[23] Teleş M. , Validity and Reliability of the Turkish Version of the General Work Stress Scale, Journal of Nursing Management. (2021) 29, no. 4, 710–720, 10.1111/jonm.13211. 33174261

[24] Karadas A. and Çevik C. , Psychometric Analysis of the Quiet Quitting and Quiet Firing Scale Among Turkish Healthcare Professionals, Journal of Evaluation in Clinical Practice. (2025) 31, no. 2, 10.1111/jep.14136. PMC11938806 39279539

[25] Galanis P. , Katsiroumpa A. , Moisoglou I. , Kalogeropoulou M. , Gallos P. , and Vraka I. , Emotional Intelligence Protects Nurses Against Quiet Quitting, Turnover Intention, and Job Burnout, AIMS Public Health. (2024) 11, no. 2, 601–613, 10.3934/publichealth.2024030. 39027384 PMC11252582

[26] Koçak D. , Kerse G. , and Yücel İ. , If Organizational Citizenship is Compulsory, Will Job Satisfaction Be Affected? A Study in the Context of Job Stress, Afyon Kocatepe University Journal of Social Sciences. (2019) 21, no. 2, 547–560, 10.32709/akusosbil.505213.

[27] Liang H.-L. , Yeh T.-K. , and Wang C.-H. , Compulsory Citizenship Behavior and Its Outcomes: Two Mediation Models, Frontiers in Psychology. (2022) 13, 10.3389/fpsyg.2022.766952. PMC8855056 35185718

[28] Ünaldı Baydın N. , Tiryaki Sen H. , Gürler S. , Dalli B. , and Harmancı Seren A. K. , A Study on the Relationship Between Nurses’ Compulsory Citizenship Behaviours and Job Stress, Journal of Nursing Management. (2020) 28, no. 4, 851–859, 10.1111/jonm.13009. 32187768

[29] Geng R. , Geng X. , and Geng S. , Identifying Key Antecedents of Quiet Quitting Among Nurses: A Cross-Profession Meta-Analytic Review, Journal of Advanced Nursing. (2026) 82, no. 1, 913–933, 10.1111/jan.16934. 40167291

[30] Yücel Y. , Workplace Stress, Work and Society Journal. (2022) 3, no. 74, 1959–1988, 10.54752/ct.1142742.

[31] Tahir M. Z. , Awan T. M. , Mughal F. , and Waheed A. , The Cascading Role of Leader-Induced Defensive Cognitions and Citizenship Pressures in Navigating Employee Silence, Management Research Review. (2024) 48, no. 1, 1–21, 10.1108/MRR-12-2023-0920.

[32] He P. , Zhou Q. , Zhao H. , Jiang C. , and Wu Y. , Compulsory Citizenship Behavior and Its Outcomes: Two Mediation Models, Frontiers in Psychology. (2022) 13, 10.3389/fpsyg.2022.766952. PMC8855056 35185718

[33] Wu M. , Peng Z. , and Estay C. , How Destructive Leadership Influences Compulsory Organizational Citizenship Behavior, Chinese Management Studies. (2018) 12, no. 2, 453–468, 10.1108/CMS-10-2017-0298.

[34] Kang J. , Kim H. , and Cho O. H. , Quiet Quitting Among Healthcare Professionals in Hospital Environments: A Concept Analysis and Scoping Review Protocol, BMJ Open. (2023) 13, no. 11, 10.1136/bmjopen-2023-077811. PMC10660974 37984954

