# Supplementary material for: Compulsory Citizenship Behavior, Work Stress, Perceived Quiet Firing, and Quiet Quitting Among Nurses: A Mediation and Exploratory Serial Mediation Analysis
Source: J Nurs Manag. 2026 Aug 17;2026:1888100. doi: 10.1155/jonm/1888100 (PMC13478906; doi:10.1155/jonm/1888100)
Supplement: Supplementary file 1 [file JONM-2026-1888100-s001.docx]

STROBE Statement—checklist of items that should be included in reports of observational studies

|  | Item No. | Recommendation | Page  No. | Relevant text from manuscript |
| --- | --- | --- | --- | --- |
| **Title and abstract** | 1 | (*a*) Indicate the study’s design with a commonly used term in the title or the abstract | P # 1 | Examining the Relationships Between Quiet Quitting, Quiet Firing, Compulsory Citizenship Behavior, and Work Stress Among Nurses  ABSTRACT |
|  |  | (*b*) Provide in the abstract an informative and balanced summary of what was done and what was found | P # 1 | A cross-sectional, model-testing design was employed. The sample consisted of 624 nurses employed at three public hospitals. Statistical analyses included Pearson’s correlation, multivariate linear regression using the stepwise method, structural equation modeling, and Bootstrap method. |
| Introduction | | | |  |
| Background/rationale | 2 | Explain the scientific background and rationale for the investigation being reported | P # 2-4 | 1.Introduction |
| Objectives | 3 | State specific objectives, including any prespecified hypotheses | P # 4 | **H_1a_:** Compulsory citizenship behavior among nurses increases quiet quitting.  **H_2a_:** Compulsory citizenship behavior increases work stress in nurses.  **H_3a_:** Work stress increases quiet quitting among nurses.  **H_4a:_** Work stress mediates the relationship between compulsory citizenship behavior and quiet quitting in nurses.  **H_1b_:** Compulsory citizenship behavior among nurses increases quiet quitting.  **H_2b_:** Compulsory citizenship behavior increases work stress in nurses.  **H_3b_:** Work stress increases quiet quitting among nurses.  **H_4b_:** Work stress mediates the relationship between compulsory citizenship behavior and quiet quitting in nurses. |
| Methods | | | |  |
| Study design | 4 | Present key elements of study design early in the paper | P # 4 | This study adopted a model-testing approach using cross-sectional data. |
| Setting | 5 | Describe the setting, locations, and relevant dates, including periods of recruitment, exposure, follow-up, and data collection | P # 5 | **Population and Sample:**  The study population consisted of 1,390 nurses employed in three public hospitals. During the study period, 827 nurses who were not on long-term leave were approached. Of these, 636 nurses agreed to participate and received the questionnaires.  **Data Collection Process:**  The study was conducted between November 30 and December 30, 2024. Data were collected through face-to-face administration of questionnaires with nurses who voluntarily agreed to participate. |
| Participants | 6 | (*a*) *Cohort study*—Give the eligibility criteria, and the sources and methods of selection of participants. Describe methods of follow-up  *Case-control study*—Give the eligibility criteria, and the sources and methods of case ascertainment and control selection. Give the rationale for the choice of cases and controls  *Cross-sectional study*—Give the eligibility criteria, and the sources and methods of selection of participants | P # 5 | Data were collected through face-to-face administration of questionnaires with nurses who voluntarily agreed to participate. |
|  |  | (*b*) *Cohort study*—For matched studies, give matching criteria and number of exposed and unexposed  *Case-control study*—For matched studies, give matching criteria and the number of controls per case | - | - |
| Variables | 7 | Clearly define all outcomes, exposures, predictors, potential confounders, and effect modifiers. Give diagnostic criteria, if applicable | - | - |
| Data sources/ measurement | 8* | For each variable of interest, give sources of data and details of methods of assessment (measurement). Describe comparability of assessment methods if there is more than one group | P # 5-7 | Data Collection Instruments |
| Bias | 9 | Describe any efforts to address potential sources of bias | - | - |
| Study size | 10 | Explain how the study size was arrived at | P # 5 | Population and Sample |

Continued on next page

| Quantitative variables | 11 | Explain how quantitative variables were handled in the analyses. If applicable, describe which groupings were chosen and why | P # 7-8 | Quantitative variables were treated as continuous variables in the analyses. Descriptive statistics are presented as mean and standard deviation. |
| --- | --- | --- | --- | --- |
| Statistical methods | 12 | (*a*) Describe all statistical methods, including those used to control for confounding | P # 7-8 | Data Analysis |
|  |  | (*b*) Describe any methods used to examine subgroups and interactions | - | - |
|  |  | (*c*) Explain how missing data were addressed | - | - |
|  |  | (*d*) *Cohort study*—If applicable, explain how loss to follow-up was addressed  *Case-control study*—If applicable, explain how matching of cases and controls was addressed  *Cross-sectional study*—If applicable, describe analytical methods taking account of sampling strategy | - | - |
|  |  | (*e*) Describe any sensitivity analyses | - | - |
| Results | | | | |
| Participants | 13* | (a) Report numbers of individuals at each stage of study—eg numbers potentially eligible, examined for eligibility, confirmed eligible, included in the study, completing follow-up, and analysed | P # 5 | During the study period, 827 nurses who were not on long-term leave were approached. Of these, 636 nurses agreed to participate and received the questionnaires. Twelve questionnaires were excluded due to incomplete responses. The final sample comprised 624 nurses who fully completed the questionnaire, representing 44.89% of the total population and 98.11% of those who initially participated. |
|  |  | (b) Give reasons for non-participation at each stage | - | - |
|  |  | (c) Consider use of a flow diagram | - | - |
| Descriptive data | 14* | (a) Give characteristics of study participants (eg demographic, clinical, social) and information on exposures and potential confounders | P # 8 | 3.RESULTS  3.1. Descriptive characteristics |
|  |  | (b) Indicate number of participants with missing data for each variable of interest | - | - |
|  |  | (c) *Cohort study*—Summarise follow-up time (eg, average and total amount) | - | - |
| Outcome data | 15* | *Cohort study*—Report numbers of outcome events or summary measures over time | *-* | *-* |
|  |  | *Case-control study—*Report numbers in each exposure category, or summary measures of exposure | *-* | *-* |
|  |  | *Cross-sectional study—*Report numbers of outcome events or summary measures | P # 7 | Cronbach’s alpha coefficients were calculated to assess the internal consistency reliability of the measurement scales used in the study. Categorical variables were summarized using frequencies and percentages (n, %), while continuous variables were presented as means and standard deviations. Pearson’s correlation analysis was conducted to examine the relationships between pairs of continuous variables. To determine the independent predictors of nurses’ perceptions of quiet quitting and quiet firing, multivariate linear regression analyses were performed using the stepwise method. Structural equation modeling (SEM) was applied to evaluate the hypothesized relationships among the study variables. |
| Main results | 16 | (*a*) Give unadjusted estimates and, if applicable, confounder-adjusted estimates and their precision (eg, 95% confidence interval). Make clear which confounders were adjusted for and why they were included | - | - |
|  |  | (*b*) Report category boundaries when continuous variables were categorized | - | - |
|  |  | (*c*) If relevant, consider translating estimates of relative risk into absolute risk for a meaningful time period | - | - |

Continued on next page

| Other analyses | 17 | Report other analyses done—eg analyses of subgroups and interactions, and sensitivity analyses | - | - |
| --- | --- | --- | --- | --- |
| Discussion | | | | |
| Key results | 18 | Summarise key results with reference to study objectives | P # 10-13 | Discussion |
| Limitations | 19 | Discuss limitations of the study, taking into account sources of potential bias or imprecision. Discuss both direction and magnitude of any potential bias | P # 13 | Limitations |
| Interpretation | 20 | Give a cautious overall interpretation of results considering objectives, limitations, multiplicity of analyses, results from similar studies, and other relevant evidence | P # 13 | Limitations |
| Generalisability | 21 | Discuss the generalisability (external validity) of the study results | P # 13 | Limitations |
| Other information | |  | | |
| Funding | 22 | Give the source of funding and the role of the funders for the present study and, if applicable, for the original study on which the present article is based | None | None |

*Give information separately for cases and controls in case-control studies and, if applicable, for exposed and unexposed groups in cohort and cross-sectional studies.

**Note:** An Explanation and Elaboration article discusses each checklist item and gives methodological background and published examples of transparent reporting. The STROBE checklist is best used in conjunction with this article (freely available on the Web sites of PLoS Medicine at http://www.plosmedicine.org/, Annals of Internal Medicine at http://www.annals.org/, and Epidemiology at http://www.epidem.com/). Information on the STROBE Initiative is available at www.strobe-statement.org.
